# Advancing the integration of multi‐marker metabarcoding data in dietary analysis of trophic generalists

**DOI:** 10.1111/1755-0998.13060

**Published:** 2019-08-26

**Authors:** Luís P. da Silva, Vanessa A. Mata, Pedro B. Lopes, Paulo Pereira, Simon N. Jarman, Ricardo J. Lopes, Pedro Beja

**Affiliations:** ^1^ CIBIO‐InBIO, Research Center in Biodiversity and Genetic Resources University of Porto Vairão Portugal; ^2^ CEF, Center for Functional Ecology ‐ Science for People & the Planet, Department of Life Sciences University of Coimbra Coimbra Portugal; ^3^ Department of Biology, Faculty of Sciences University of Porto Porto Portugal; ^4^ Erada Portugal; ^5^ School of Biological Sciences University of Western Australia Perth WA Australia; ^6^ Environomics Future Science Platform CSIRO National Collections and Marine Infrastructure Crawley WA Australia; ^7^ CIBIO‐InBIO, Research Center in Biodiversity and Genetic Resources, Institute of Agronomy University of Lisbon Lisbon Portugal

**Keywords:** bird, diet, metabarcoding, morphological identification, overlapping markers, secondary predation

## Abstract

The application of DNA metabarcoding to dietary analysis of trophic generalists requires using multiple markers in order to overcome problems of primer specificity and bias. However, limited attention has been given to the integration of information from multiple markers, particularly when they partly overlap in the taxa amplified, and vary in taxonomic resolution and biases. Here, we test the use of a mix of universal and specific markers, provide criteria to integrate multi‐marker metabarcoding data and a python script to implement such criteria and produce a single list of taxa ingested per sample. We then compare the results of dietary analysis based on morphological methods, single markers, and the proposed combination of multiple markers. The study was based on the analysis of 115 faeces from a small passerine, the Black Wheatears (*Oenanthe leucura*). Morphological analysis detected far fewer plant taxa (12) than either a universal 18S marker (57) or the plant *trn*L marker (124). This may partly reflect the detection of secondary ingestion by molecular methods. Morphological identification also detected far fewer taxa (23) than when using 18S (91) or the arthropod markers IN16STK (244) and ZBJ (231), though each method missed or underestimated some prey items. Integration of multi‐marker data provided far more detailed dietary information than any single marker and estimated higher frequencies of occurrence of all taxa. Overall, our results show the value of integrating data from multiple, taxonomically overlapping markers in an example dietary data set.

## 1 INTRODUCTION

Studies on trophic interactions using next generation sequencing (NGS) approaches have had an increasing impact on ecological research (Bohmann et al., 2014; Deiner et al., 2017; Taberlet, Bonin, Zinger, & Coissac, 2018; Taberlet, Coissac, Pompanon, Brochmann, & Willerslev, 2012), revolutionizing the breadth and depth of dietary studies, making it possible to process hundreds or even thousands of samples in a relatively short time (Galan et al., 2018; Nielsen, Clare, Hayden, Brett, & Kratina, 2017; Pompanon et al., 2012). Furthermore, metabarcoding makes it possible to identify virtually all species consumed by a predator or herbivore, including rare food items (Hope et al., 2014; Nielsen et al., 2017; Razgour et al., 2011; Soininen et al., 2009), though this is conditional on DNA quality and the availability of DNA reference databases (Deagle, Eveson, & Jarman, 2006; Elbrecht et al., 2016; Gerwing, Kim, Hamilton, Barbeau, & Addison, 2016). Due to its strengths and cost‐effectiveness, this approach has been increasingly used to describe the diet of many animals (Deagle et al., 2019; Kaunisto, Roslin, Sääksjärvi, & Vesterinen, 2017; Macías‐Hernández et al., 2018; Soininen et al., 2009) and even carnivorous plants (Littlefair, Zander, Sena Costa, & Clare, 2019). However, there are still significant uncertainties regarding potential biases and pitfalls of metabarcoding, and how best to address them, which may significantly impact on the results of dietary analysis (Nielsen et al., 2017).

One problem that has attracted much attention is the selection of molecular markers, because primer specificity and biases can greatly affect the results of dietary studies (Alberdi et al., 2019; Taberlet et al., 2018). In general, studies build on previous knowledge of the diet of one or more species of interest, or of ecologically similar species, to select a primer that amplifies DNA from the main food items expected to be consumed (Alberdi et al., 2019; Coghlan et al., 2013). For instance, the studies of Soininen et al. (2009) and Valentini et al. (2009) used primers amplifying a fragment of the chloroplast *trn*L intron to analyse the diet of a number of herbivore species. Likewise, many studies on insectivore diets often used the ZBJ primer amplifying a fragment of the COI mitochondrial gene (Razgour et al., 2011; Zeale, Butlin, Barker, Lees, & Jones, 2011). This single marker approach has been widely used in many studies (Gordon et al., 2019; McClenaghan, Nol, & Kerr, 2019; Moran, Prosser, & Moran, 2019), but it may produce significant biases due to differential primer affinity for different taxa. For instance, although ZBJ is often used as a ‘universal’ marker for arthropods (Crisol‐Martínez, Moreno‐Moyano, Wormington, Brown, & Stanley, 2016; Jedlicka, Vo, & Almeida, 2017; Trevelline, Latta, Marshall, Nuttle, & Porter, 2016; Trevelline et al., 2018), it may have strong positive or negative bias depending on the taxa (Clarke, Soubrier, Weyrich, & Cooper, 2014; Piñol, Mir, Gomez‐Polo, & Agustí, 2015). The challenge is even worse in the case of omnivorous diets, because the variety of taxonomic clades consumed cannot be analysed using a single marker (De Barba et al., 2014; Taberlet et al., 2018). Therefore, it is increasingly recognised that molecular dietary studies should be based on a mix of markers that adequately amplify the full complement of prey ingested, which requires integration of data from several markers for each sample (Alberdi et al., 2019; Alberdi, Aizpurua, Gilbert, & Bohmann, 2017; Deagle, Kirkwood, & Jarman, 2009; Taberlet et al., 2018).

In multi‐marker dietary studies, the most common approach is to divide the expected diet in various components (e.g., vascular plants, cephalopods, arthropods and vertebrates), and then use a primer designed to target each component (Coghlan et al., 2013; Groom, White, Mitchell, Roberts, & Mawson, 2017; Robeson et al., 2018; Sullins et al., 2018). The integration of this type of multi‐marker data is relatively straightforward, as information from each dietary component is retrieved from a single marker, and so a list of taxa detected in each sample can be inferred simply by adding taxa lists across markers. However, in some cases it may be necessary to use a mix of primers overlapping in the range of taxa amplified, making data integration more difficult. For instance, in dietary analysis of trophic generalists it may be useful to combine a universal marker with more specific markers, to account for the consumption of unexpected taxa that are not adequately detected by any of the specific primers used (De Barba et al., 2014; Deagle et al., 2009; Taberlet et al., 2018). Also, in dietary analysis involving highly diverse prey groups such as arthropods it may be necessary to avoid biases by combining primers that vary in affinity for different orders or even families, but that may overlap considerably in the range of taxa amplified (Aizpurua et al., 2018; De Barba et al., 2014; Kaunisto et al., 2017). Integration of such data cannot be made simply by adding the taxa lists retrieved across markers, because the same individual prey may be detected at different taxonomic levels by different markers, due to differences in taxonomic resolution or in the availability of reference databases (Elbrecht et al., 2016). To combine such data, it is necessary to identify duplications across markers, and to retain in each case the most taxonomically resolved taxa. Although these approaches based on taxonomically overlapping markers may advance dietary studies of trophic generalist species by maximising the diversity of species detected, they remain underutilised, there are no well‐established criteria for integrating data across markers, and there is no simple computation procedure to implement such criteria.

Here, we test the use of multiple overlapping markers, the criteria for integrating data from them in dietary analysis of a trophic generalist bird, and provide a python script to implement our data integration scheme. Prey remains retrieved from Black Wheatear (*Oenanthe leucura*) faeces were identified morphologically and using DNA metabarcoding with four molecular markers. Molecular data was integrated by the means of a python script to provide a single list of taxa detected per sample, controlling for duplications by collapsing less resolved taxa detected by one marker (e.g., order and family level) with higher resolved taxa detected using a different marker (e.g., genus and species). We then evaluated differences between morphological, single marker and multi‐marker approaches in the estimates of dietary descriptors, in terms of (a) taxonomic resolution, (b) diet diversity, (c) the identity of taxa recorded, and (d) the composition of diet considering the taxa recorded and their representation in the samples.

## 2 MATERIALS AND METHODS

### 2.1 Study species and sample collection

The Black Wheatear is a small (~35 g) black and white passerine that occurs in cliffs and rocky slopes of arid areas in western North Africa and Iberia. Although the species is not globally threatened, European populations are steadily declining, and the species is now regionally vulnerable (BirdLife International, 2017). Black Wheatears have a very diverse diet, feeding on freshly fruits, insects, arachnids, centipedes and sometimes even lizards (Hodar, 1995; Prodon, 1985; Richardson, 1965). The wheatear is a good study system to test our methodology due to its large feeding spectrum, including both plants and animals, and thus allowing us to test many food items simultaneously, and serving as model for other generalist terrestrial vertebrates. We collected 115 faecal samples from 143 Black Wheatears captured with spring‐traps baited with Mealworms (*Tenebrio molitor*) throughout their known distribution in the Douro Valley in Portugal (Figure S1), during spring and summer of 2014–2016. All birds were ringed to allow for individual recognition, which indicated that only four samples resulted from re‐trapped individuals, two from birds collected 3 months apart, and two from birds collected in different years. Faecal samples were collected from clean cotton bags (soaked in 10% bleach for 1 hr and then washed between each use) or directly from stones used to disguise the bottom of the spring‐traps (McInnes et al., 2017; Oehm, Juen, Nagiller, Neuhauser, & Traugott, 2011). Samples were stored in 98% ethanol and refrigerated at 4ºC until processed in the laboratory.

### 2.2 Molecular analysis

DNA was extracted from each faecal sample using the Stool DNA Isolation Kit (Norgen Biotek Corporation) following the manufacturer's protocol. Samples were extracted in batches of 23 plus a negative control in which no faecal material was added. After DNA extraction the remaining faecal fragments used for DNA extraction were preserved for morphological identification. This was possible because morphological identification was based on hard faecal fragments such as chitinous body parts of invertebrates, vertebrate bones, and plant seeds and epidermis, which were not destroyed by the extraction method, as assessed through visual comparison of extracted and nonextracted samples.

Four different marker sets were used to analyse the diet. A universal eukaryote 18S marker (Jarman et al., 2013); two arthropod markers: a modified version of IN16STK (Kartzinel & Pringle, 2015) for the 16S region in which some degenerate bases were added to increase the affinity of the primers (IN16STK‐1F_mod: 5′‐TRAACTCARATCAYGTAA‐3′, IN16STK‐1R_mod: 5′‐TTAGGGATAACAGCRTWA‐3′) and ZBJ (Zeale et al., 2011) for COI region; and finally the gh plant specific marker for the *trn*L intron (Taberlet et al., 2007). All primers were modified to contain Illumina adaptors at the 5′ end of the sequence (forward primers: 5′‐TCGTCGGCAGCGTCAGATGTGTATAAGAGACAG‐3′, reverse primers: 5′‐GTCTCGTGGGCTCGGAGATGTGTATAAGAGACAG‐3′). PCR reactions were carried‐out in volumes of 10 μl, comprising 5 μl of QIAGEN Multiplex PCR Master Mix, 0.3 μl of each 10 mM primer, 3.4 μl of ultra‐pure water, and 1 μl of DNA extract. Cycling conditions used initial denaturing at 95°C for 15 min, followed by 35 cycles of denaturing at 95°C for 30 s, annealing at 45°C for 30 s and extension at 72°C for 30 s, with a final extension at 72°C for 10 min. Each marker was amplified in an independent PCR reaction, without any multiplexing. A very low PCR amplification temperature was used for all markers in order to reduce as much as possible the level of primer bias, this way allowing primers to anneal with less matching templates. This has been tested for some COI markers with positive results (Clarke et al., 2014). We also did not do any PCR replicates because recent studies have shown that, for faecal samples, variation in prey species composition among PCR replicates is much smaller than variation among samples (Mata et al., 2019). Amplification success was checked by visually inspecting 2 μl of each PCR product on a 2% gel stained agarose (GelRed Biotium). PCR products were subjected to a second round of PCR with P5 and P7 indexes, after an initial dilution of 1:4 in order to reduce the amount of initial template and guarantee the complete incorporation of indexes in the library. Each index contained a unique 7 bp long barcode that differed at least 3 bp from any other index, allowing for the multiplex of several hundred samples in a single run (P5: 5′‐AATGATACGGCGACCACCGAGATCTACACxxxxxxxTCGTCGGCAGCGTC‐3′, P7: 5′‐CAAGCAGAAGACGGCATACGAGATxxxxxxxGTCTCGTGGGCTCGG‐3′). PCR reactions and cycling conditions were similar to the ones of the first PCR except that only 8 cycles of denaturing, annealing and extension were done, with annealing at 50ºC. PCR products were purified using Agencourt AMPure XP beads (Beckman Coulter), and subsequently quantified using Nanodrop and diluted to 15 nM. Purified and normalized PCR products were pooled per marker. These four libraries were then individually quantified using qPCR (KAPA Library Quant Kit qPCR Mix; Bio‐Rad iCycler) and diluted to 4 nM. Finally, libraries were pooled equimolarly and sequenced using approximately half a lane of a 500 cycles v2 MiSeq run (Illumina) for an expected average of 24,000 paired‐end reads per sample‐marker combination.

### 2.3 Bioinformatic analysis

Bioinformatic processing of sequencing reads was done using OBITools (Boyer et al., 2016), with a separate analysis for each molecular marker. First, paired‐end reads were aligned using the command ‘illuminapairedend’ and discarded if overlapping quality was <40 (Taberlet et al., 2018). Second, reads were assigned to samples and primer sequences were removed using ‘ngsfilter’, allowing a total of four mismatches to the expected primer sequence. Finally, reads were collapsed into exact sequence variants (ESVs) and singletons were removed. ESV diversity and read count per fragment length, as well as bibliographic information of each marker was used to discard ESVs shorter and/or longer than expected. This way, we kept fragments with 94–153 bp for 18S, 72–119 bp for IN16STK, 155–159 bp for ZBJ, and 30–93 bp for *trn*L. The command ‘obiclean’ was then used to denoise the data by removing potentially spurious sequences with an ‘r’ level of one. This means that any ‘A’ ESV differing one base‐pair from a ‘B’ ESV, with an absolute read count lower than ‘B’, and that was not found without the presence of ‘B’ in any PCR product, was removed as it was most likely a PCR or sequencing error. The PCR products that exhibited less than 100 reads in total after this step were considered to have failed and excluded from further analyses. This only happened for negative controls and taxa specific markers (IN16STK, ZBJ, and *trn*L), meaning that all samples contained amplifiable DNA. For the remaining ones, we removed from each PCR product all ESVs that had a read count <1% of the total number of reads of that PCR (Mata et al., 2019). This should allow the removal of most PCR and sequencing errors that still passed the ‘obiclean’ denoising step.

For each marker, prey items were identified by comparing the ESVs retained against online databases (BOLD and NCBI) using BLAST algorithm, as well as unpublished sequences of 1,846 species of arthropods collected in northern Portugal in the case of COI (for further details see Ferreira et al., 2018). Whenever an ESV matched several species, genus, or families at similar identity levels, we selected the most inclusive taxonomic rank. For example, if a given 16S ESV matched with 99% similarity to two species of different genus belonging to the same family, we identified that ESV only to family level. For ESVs not identified to species level, we built a neighbour‐joining tree in Geneious (Biomatters), visually inspected the corresponding alignment, and checked for patterns of co‐occurrence of similar ESVs in order to cluster (~98%) them into distinct taxa (e.g., Carabidae 1, Carabidae 2, and so on), also referred as molecular operational taxonomic units (MOTUs). After this step, we removed every taxa not belonging to either the Plantae or Animal kingdoms, as well as all nonvascular plants, birds (mostly ESVs matching Black Wheatear), mammals (human and pig), internal parasites (phylum Nematoda), as well as mealworms (and the only Tenebrionidae MOTU found with 18S and assumed to be mealworm) due to the high probability of being bait contamination. In the end, for each marker we counted the total number of taxa identified in each sample at the highest possible taxonomic resolution, thereby summing the number of taxa identified at species level with other MOTUs identified at higher taxonomic categories.

To build a consensus diet incorporating all molecular markers, we developed a python 3.0 script that merges the dietary information derived from the four markers into a single taxa list per sample. The script functions by merging in each individual sample the different taxa obtained with the different markers, considering the differences in taxonomic resolution yielded by different markers. This merging assumed that a given item recovered at higher taxonomic resolution (e.g., order or family) by a given marker was the same as items of the same taxonomic group recovered at lower resolution by other markers (e.g., genus or species). For example, if in a given sample the 18S marker detected a Coleoptera, the IN16STK a Chrysomelidae, and ZBJ a species belonging to the Chrysomelidae family, we assumed that all the markers were detecting the same taxa and merged them all into the most taxonomically resolved taxa. In contrast, we assumed the presence of different items when taxonomy at different levels of resolution was inconsistent across markers. For instance, if the 18S detected a Coleoptera, IN16STK a Carabidae, and ZBJ a species belonging to the Chrysomelidae family, we assumed there were two distinct taxa: the Carabidae and the Chrysomelidae species. This was expected to enhance complementarities and avoid redundancies across markers. However, since for many MOTUs it is impossible to establish a clear taxonomic relationship between the different markers, due to different taxonomic resolutions and lack of clear co‐occurrences, we opted to merge MOTUs only on a sample by sample basis. For instance, MOTU‐1 from 18S identified as undetermined Coleoptera1 could be merged in one sample with MOTU‐2 from IN16STK identified as undetermined Carabidae1, but in a different sample could be merged with MOTU‐3 identified as undetermined Chrysomelidae1. This could happen because the different families of beetles could share the same 18S MOTU, but also because different taxa are being detected with each marker. However, since there is no way to distinguish both situations, we believe our merging approach to be conservative and to avoid overestimating dietary diversity. Taxa richness per sample was computed as for the individual markers, by counting the total number of taxa identified at the highest possible taxonomic resolution. The python code is provided in Supporting Information (merge_script.rar) with a ‘readme’ explanatory file containing an example of data input, and will be made available at the GitHub Repository upon manuscript acceptance.

### 2.4 Morphological identification

Plant and animal remains from faecal samples were analysed under a dissecting microscope, except plant epitheliums that were seen under a compound microscope, after DNA extraction. Plant remains like seeds and epidermis were identified by comparison with plants collected at capture sites. Animal parts were identified to the order or family level whenever possible, using specialized bibliography (Barrientos, 2004). In each sample, we also identified the total number of animal morphospecies of each order, thereby producing an approximation to the total number of taxa per sample. We did not, however, compare morphospecies across all samples in order to estimate the total diversity, because they were rarely comparable due to differences in the fragments recorded in each sample. This should not affect the analysis as all comparisons with molecular data were done on a sample by sample basis.

### 2.5 Data analysis

Statistical analysis was conducted to detect significant variation in estimates of dietary descriptors (i.e., diet diversity and composition) between different molecular markers, and to compare estimates obtained with each individual marker and the multi‐marker approach. As multi‐marker data combines information from all individual markers, it was used as the benchmark against which the performance of each individual marker was compared. This allowed, for instance, to assess what taxa are consistently missed or underestimated by the single markers. Plant and animal components of the diet were always analysed separately due to the different taxonomic range of the primers. Statistical significance was considered for *p*‐values ≤.05. All analysis were carried in r v3.3.0 (R Core Team, 2016).

To evaluate whether there were differences between methods in diet diversity estimates, for both plant and animal components, we compared among methods (a) the numbers of taxa per sample, and (b) the number of orders per sample, using generalized linear mixed models (GLMM) with a Poisson distribution and a log link, specifying the sample as random effect. GLMMs were performed using the packages lme4 (Bates, Mächler, Bolker, & Walker, 2015) and lmertest (Kuznetsova, Brockhoff, & Christensen, 2017). We then used multi‐comparisons with Bonferroni corrections to identify in which pairs the observed differences occurred, using the package multcomp (Hothorn, Bretz, & Westfall, 2008). To evaluate differences between methods in the estimates of diet composition, we used Multivariate Generalized Linear Models, assuming negative binomial errors, with the package mvabund (Wang, Naumann, Wright, & Warton, 2012). Analysis were carried out using numbers of taxa of each order detected per sample as response variable. To detect which orders contributed to differences among methods we used univariate tests with adjusted *p*‐values for multiple testing. Finally, we used Czekanowski's overlap index (Nielsen et al., 2017) to estimate the pairwise overlap in diet composition estimated by different methods, using the r package ecosimr (Gotelli, Hart, & Ellison, 2015). This index ranges from 0 (no overlap) to 1 (complete overlap) and compares pairwise similarities based on frequency of occurrence data.

## 3 RESULTS

### 3.1 Plant component

**Figure 1:**
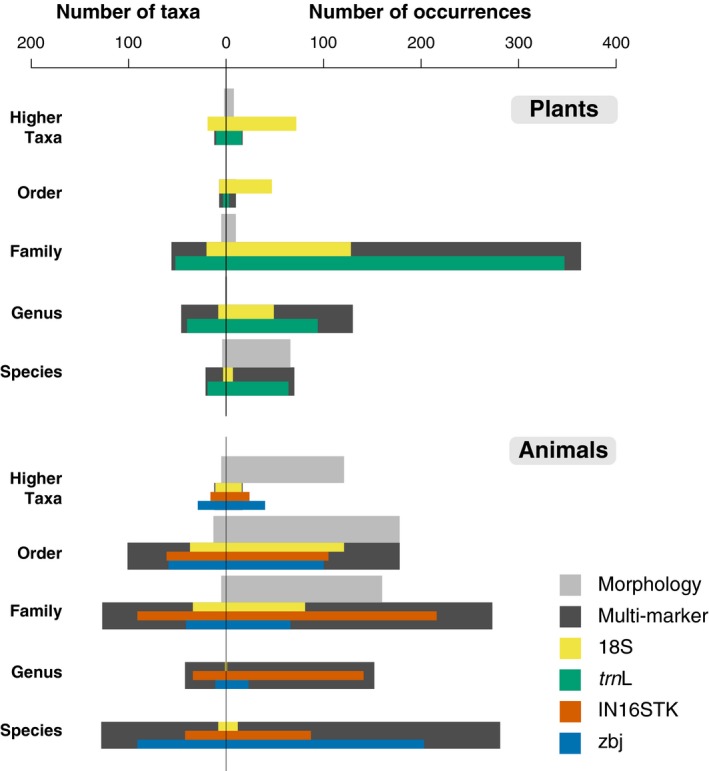
Number of consumed taxa observed at different taxonomic levels (left) and number of occurrences observed at each taxonomic level (right), during the morphological identification (Morphology), with four individual molecular markers (18S, universal marker; *trn*L, plant marker; IN16STK and ZBJ, arthropod specific markers) and with the multi‐marker approach, for plants and animals. Note that for the morphological identification, animal fragments were not compared across samples, and therefore the total number of taxa corresponds to the sum of the maximum number of morphotypes detected per family and order [Colour figure can be viewed at http://wileyonlinelibrary.com]

**Figure 2:**
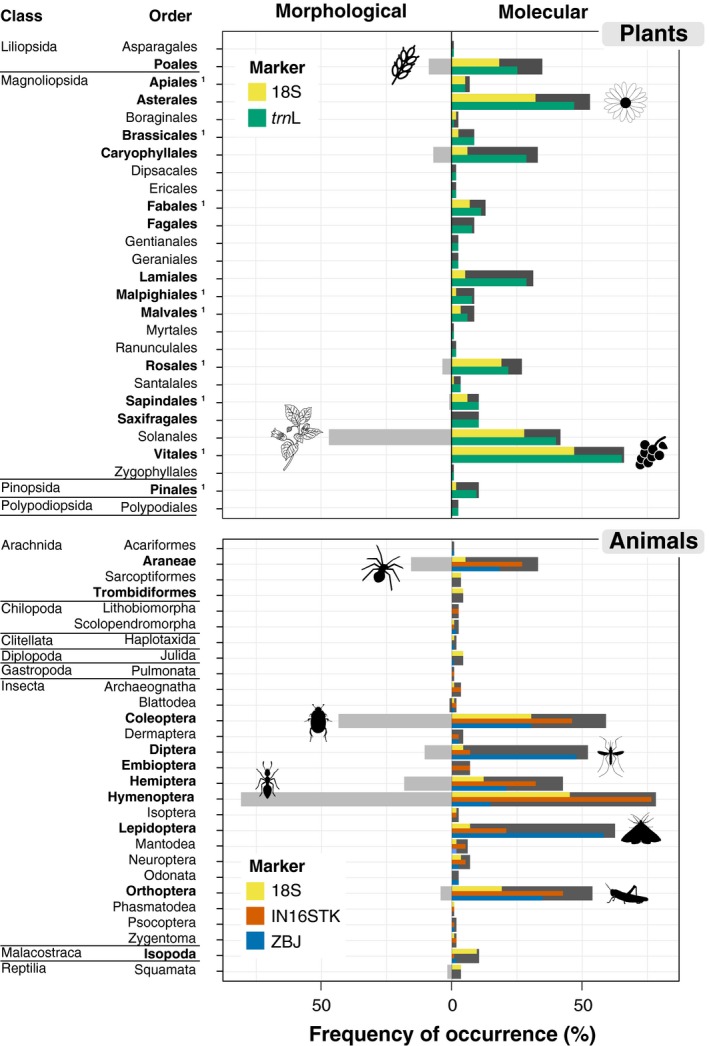
Frequencies of occurrence of each order of plants and animals in the diet of Black Wheatears obtained through morphological and molecular analysis (multi‐marker, dark grey bar, and for each set of primers). The orders highlighted in bold indicate significant differences at univariate tests of Multivariate Generalized Linear Models. ^1^indicates orders that only showed significant differences among the molecular markers and morphological identification [Colour figure can be viewed at http://wileyonlinelibrary.com]

**Figure 3:**
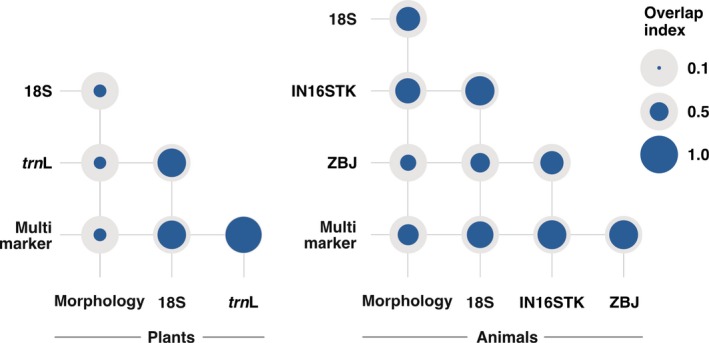
Czekanowski's overlap index for plants and animals, between the morphological identification, the several molecular markers and the multi‐marker approach used in Black Wheatear diet analysis [Colour figure can be viewed at http://wileyonlinelibrary.com]

Morphological examination detected plants in 73 out of 115 faecal samples, yielding 12 taxa from five orders, of which five taxa were identified to genus or species levels (Figure 1). The most frequent taxon was *Solanum nigrum*, order Solanales (Figure 2). Metabarcoding detected plants in more faecal samples and yielded more taxa than morphology using either 18S (100 samples; 57 taxa from 16 orders; 2,479 ± 220 reads/sample) or *trn*L (110 samples; 124 taxa from 27 orders; 7,462 ± 387 reads/sample) (Figure 2). Besides detecting almost twice as many taxa, the taxonomic resolution was much higher for *trn*L (54% of taxa identified to genus or species) than 18S (19% to genus or species; Figure 1). The taxa recorded most frequently using either 18S or *trn*L was an unidentified plant of the family Vitaceae, most probably *Vitis vinifera* (Figure 2). There was significant variation among methods in the number of orders (*χ* ^2^ = 200.77, *df* = 2, *p* < .001) and taxa (*χ* ^2^ = 289.58, *df* = 2, *p* < .001) detected, with much lower values for morphology than metabarcoding with either 18S or *trn*L (Table 1; Table S1). There were also significant differences in plant composition between methods (Wald value = 11.21, *p* < .001), with univariate tests indicating that 15 plant orders, particularly Vitales and Asterales, significantly contributed to such differences (Figure 1; Table S2). Overlap was high between the results of 18S and *trn*L (0.757), but each had low overlap with morphology (<0.350; Figure 3).

**Table 1:** Average ± standard error of the number of orders and taxa detected per sample

	Method	Order	Taxa
Plant	Morphological	1.05 ± 0.05	1.12 ± 0.04
	18S	2.14 ± 0.11	3.03 ± 0.17
	*trn*L	3.70 ± 0.18	4.76 ± 0.31
	Multi‐marker	4.09 ± 0.19	5.30 ± 0.31
Animal	Morphological	1.80 ± 0.10	4.09 ± 0.20
	18S	1.91 ± 0.12	2.38 ± 0.16
	IN16STK	2.93 ± 0.13	5.07 ± 0.27
	ZBJ	2.58 ± 0.12	4.00 ± 0.20
	Multi‐marker	4.56 ± 0.17	7.92 ± 0.32

Note

The number of taxa combines the number of species identified and the number of MOTUs identified at higher taxonomic levels.

### 3.2 Animal component

Morphological examination detected animal prey in 112 samples, yielding 23 taxa from eight orders, all of which were identified at best to family level (Figures 1 and 2). The most frequent order was Hymenoptera (81%), mainly due to the family Formicidae (70%; Figure 2). Metabarcoding using 18S detected animals in 94 samples (3,008 ± 299 reads/sample), yielding 91 taxa from 21 orders, of which 10% were assigned to a genus or a species (Figures 1 and 2). The most frequent order was also Hymenoptera (45%). The two arthropod specific markers, IN16STK and ZBJ detected animals in 113 (6,765 ± 342 reads/sample) and 108 (2,829 ± 202 reads/sample) samples, yielding 244 and 231 taxa from 21 and 18 orders, respectively. From the taxa identified, 31% and 42%, IN16STK and ZBJ respectively, were identified to genus or species (Figure 1). The most frequent order detected by IN16STK was Hymenoptera (77%), mainly due to Formicidae (71%) as in the morphological analysis, while ZBJ detected most frequently Lepidoptera (59%) and only detected Hymenoptera in 15% of the samples, failing to detect the family Formicidae (Figure 2).

The mean number of taxa per sample varied significantly among the morphological identification and the markers (*χ* ^2^ = 148.78, *df* = 3, *p* < .001), with all differing significantly from each other, except morphology from ZBJ (Table 1; Table S1). Likewise, there was significant variation in the mean number of orders per sample across methods (*χ* ^2^ = 54.78, *df* = 3, *p* < .001), with all differing significantly from each other, except morphology from 18S, and IN16STK from ZBJ (Table 1; Table S1). Finally, there were significant differences in animal composition among morphological and molecular methods (Wald value = 21.29, *p* < .001), with univariate tests indicating that 10 orders, particularly Hymenoptera, Lepidoptera and Orthoptera, significantly contributed to such differences (Figure 1; Table S3). Overlap between morphology and each molecular marker (0.435–0.673) was only slightly lower than the pairwise overlap between markers (0.525–0.781; Figure 3).

### 3.3 Multi‐marker approach

When integrating information from the four molecular markers used, the initial 2,064 occurrences (828 plant and 1,236 animal) were reduced to 1,492 (591 plant and 901 animal), indicating that only approximately one quarter of the information provided by the individual markers was redundant. The multi‐marker approach detected a total of 27 plant and 28 animal orders, in 112 and 115 samples, respectively. The most detected plant order was Vitales, and the most detected animal order was Hymenoptera (Figure 2). As expected, individual markers differed significantly from each other and from the multi‐marker approach in terms of taxa detected for both plants (*χ* ^2^ = 142.43, *df* = 2, *p* < .001) and animals (*χ* ^2^ = 444.93, *df* = 3, *p* < .001). The multi‐marker approach provided more occurrences with high taxonomic resolution, i.e., genus or species, (Figure 1) and also detected a higher number of taxa and orders per sample than any individual marker, except for *trn*L that contributed to most of the plant taxa present in the multi‐marker approach (Table 1; Table S1). The overlap of the multi‐marker data for the plant component was very high in relation to *trn*L (0.959) and very low in relation to morphology (0.342), while regarding the animal component the overlap was lowest with morphology (0.563) and had similarly high values with each individual marker (0.711–0.777; Figure 3).

Finally, plant and animal composition differed among the multi‐marker approach and individual markers (plants: Wald value = 10.53, *p* < .001; animals: Wald value = 22.58, *p* < .001). For plants, univariate tests, adjusted for multiple testing, indicated that these differences were caused by six orders, mainly Caryophyllales, Lamiales, and Saxifragales (Table S2). For animals, these differences were caused by 10 orders, mainly Diptera, Lepidoptera and Hymenoptera (Table S3).

## 4 DISCUSSION

Our study highlights the challenges involved in the description of the diet of trophic generalist animals, showing that results greatly vary depending on the method used. As expected, there were major differences in estimates of diet diversity, prey taxonomic identity, and composition between morphological and molecular methods, but there were also large variations in the results produced using different molecular markers. In particular, we found that widely used markers consistently underrepresented or missed some heavily consumed taxa, including taxa that were easily detected using the morphological analysis. The multi‐marker approach appeared to largely overcome the problems of underestimate biodiversity that single marker dietary or nonmolecular analysis produce, though it shares problems such as the detection of secondary ingestion. Overall, we suggest that using a mix of universal eukaryote and more taxon‐specific markers can advance the description of trophic generalist diets and underline the importance of adequately integrating data to overcome problems associated with different taxonomic resolution across markers.

### 4.1 Biases and pitfalls in morphological and molecular dietary data

Most plant material recovered visually from Black Wheatear faeces were seeds of berry‐producing plants, mainly *S. nigrum* and, to a much lesser extent, *Periplaneta americana*. These results suggest that wheatears regularly consumed berries in our study area, more so than suggested by previous studies (Hodar, 1995; Prodon, 1985; Richardson, 1965). Surprisingly, metabarcoding showed an even greater consumption of plants, with 18S and particularly *trn*L detecting a very large diversity of taxa, most of which produce dry seeds rather than berries. Reasons for this are unknown, but it may be a consequence of several nonexclusive factors. One possibility is that metabarcoding detects direct consumption of items for periods longer than the defaecation time (Deagle, Chiaradia, McInnes, & Jarman, 2010; Oehm et al., 2011), especially if short amplicons are used (Kamenova et al., 2018). This can explain for instance, why *trn*L detected the berry‐producing *Pistacia terebinthus* in 11 samples, while the seeds of this plant were detected in a single faecal sample. Another hypothesis is that the method is detecting plants that left no hard parts, and thus could not be detected visually. Lack of seeds can occur when wheatears only eat the flesh of berries, which might explain the high prevalence of *V. vinifera* detected through metabarcoding but not visually. However, this is questionable because grapes at the time of sampling were unripe and thus unlikely to be eaten by the birds. The typical insectivore morphology and behaviour of Black Wheatears (Richardson, 1965) also question the hypothesis of direct consumption to explain the detection of DNA from species with small and dry seeds such as Asterales, Lamiales and Poales, or with large acorns such as oaks *Quercus* spp. It is also highly implausible that wheatears are feeding on other parts of these plants such as buds, flowers, or pollen. A more likely explanation may thus be indirect consumption through the stomach contents of animal prey, which may be recovered by molecular markers amplifying small DNA fragments (<200 bp), such as the 18S and *trn*L markers used in our study (Kamenova et al., 2018; Sheppard et al., 2005). Detection of secondary consumption is well documented even through traditional methods (Johnson, Ross, & Smith, 1997), but it is usually considered as having little importance (Barrett et al., 2007). In metabarcoding diet studies the effect of secondary consumption is not often explored in detail, and depending on the studied species it is considered to have low impact (Gerwing et al., 2016) or considerable influence on the range of the species detected in the diet (Bowser, Diamond, & Addison, 2013). If we consider only plants likely to be directly eaten by the wheatear, i.e., with fleshy fruits ripe during the sampling period, we will only retain 8.7% and 8.0% of the plants identified by the 18S and *trn*L, respectively. This shows that secondary detection can cause a strong bias on inferring the diet of generalist vertebrates if other sources of information such as morphological analysis and behavioural studies are not used to differentiate between primary and secondary consumption. Wheatears may also accidentally ingest some plant material when capturing small prey, as suggested by the small Poales seeds found in the morphological analysis. Also, it cannot be ruled out the possibility that some of the plant DNA recovered from faeces reflects environmental contamination, including for instance contamination with pollen spread through the air. We believe, however, that these problems should have had limited impact in our results, because most samples were collected from clean bags in which environmental contamination should be minimum, and we have followed the established protocols to minimize direct contamination (McInnes et al., 2017). Finally, it is possible that the high detection of secondary ingestion was particularly high in a largely carnivore species such as wheatears, because in many faecal samples there were no remains of plant material ingested directly, and so the primers amplified the only available plant DNA, i.e., meals of herbivorous insect prey. Whatever the reasons, our results suggest that dietary metabarcoding may record DNA of many plants that are not directly ingested by the target species.

The animal prey detected visually in Black Wheatear faeces was in line with previous studies (Hodar, 1995; Prodon, 1985; Richardson, 1965), showing a prevalence for Hymenoptera, mainly Formicidae, and Coleoptera, Hemiptera, Araneae and Diptera. As expected, these groups were largely recovered through molecular analysis, though metabarcoding yielded a much larger diversity of prey and higher taxonomic resolution, particularly in the case of the COI marker ZBJ (Hope et al., 2014; Krüger, Clare, Greif, et al., 2014; Krüger, Clare, Symondson, Clare, Symondson, Keišs, & Pētersons, 2014; Razgour et al., 2011). Furthermore, some taxa were far more often detected through metabarcoding than by visual examination, including orders that seemed to be important in the diet such as Lepidoptera and Orthoptera. This may be a consequence of the ingestion of soft‐bodied animals leaving few or no hard parts (Nielsen et al., 2017; Sutherland, Newton, & Green, 2004), as it was probably the case of caterpillars (Lepidoptera). Lack of Orthoptera remains are more difficult to explain because they have a heavy chitinous exoskeleton, but this may be a consequence of wheatears eating only the soft parts of the abdomen and leaving the head, thorax and legs, thereby ingesting fewer hard parts with morphological taxonomic value.

Although we cannot rule out the possibility of some animal prey detected through metabarcoding but not morphology, such as orthoptera and other taxa, being the result of secondary predation, this seems highly unlikely as it is congruent with what is known of the wheatears feeding behaviour. However, where no other sources of dietary information are available, it might be impossible to distinguish primary from secondary ingestion. On the contrary, some taxa are easily recognized as nondietary items, due to their very small size and parasitic nature. For example, mites of the orders Acariformes, Trombidiformes, and Sarcoptiformes, detected through metabarcoding but not through visual examination, were probably not directly preyed by wheatears. These may have been ingested indirectly through the stomach contents of arthropod predators (Sheppard et al., 2005), or as parasites occurring in the body of arthropod prey or the birds themselves (Di Prisco et al., 2016; Gerwing et al., 2016; Martinho, Tenreiro, Ferreira, Faísca, & da Silva, 2017). Nonetheless, detection caused by secondary predation of animal prey appeared to be lower than that detected for plants. The reasons for this are not totally clear but may at least partly be explained by the very small size of the amplicon used for plants, which might have detected very small fragments of DNA originating from arthropod stomach contents.

Some animal preys were easily detected visually but not by some molecular markers. Formicidae, in particular, were often detected in faeces, while they were missed altogether by ZBJ. This was probably a consequence of the well‐known positive bias of ZBJ towards Diptera and Lepidoptera, at the expenses of other arthropod orders (Clarke et al., 2014). Although the failure to detect Formicidae was solved when using 18S and IN16STK, these tended to provide a lower taxonomic resolution of prey items, particularly in the case of Lepidoptera for which there was a very comprehensive reference database of COI barcodes. Therefore, only the combination of the three markers provided a detailed description of the animal component of Black Wheatear's diet.

### 4.2 Implications to describing diets with multi‐marker approaches

Overall, the combination of visual and molecular approaches used in this study highlighted two important sources of potential errors in the analysis of trophic generalist diets and provided some clues on how to address these problems. First, our study suggests that morphological examination and/or previous ecological information may be important in order to detect unexpected biases and pitfalls of molecular methods, providing a basis to interpret and eventually correct results. This is highlighted by the detection of a range of animal and plant taxa that probably resulted from secondary ingestion or contamination, which may be a widespread problem in molecular analysis of trophic generalists, particularly when using small amplicons such as gh for plant *trn*L (Groom et al., 2017; Liu et al., 2018; Sullins et al., 2018) or generalist molecular markers (Bowser et al., 2013). This problem might be important, for instance, in conservation studies aiming to assess key trophic resources for a given species (Groom et al., 2017; Liu et al., 2018), in behavioural ecology research (Aizpurua et al., 2018; Quéméré et al., 2013), and even when reconstructing trophic networks from molecular data (Evans, Kitson, Lunt, Straw, & Pocock, 2016). To address this problem, visual analysis of a subset of samples would be desirable (Haarsma, Siepel, & Gravendeel, 2016), providing information on the range of taxa that are eaten, which could then be compared against the results of metabarcoding. As this may often be impractical, researchers should at least check their metabarcoding results against the literature on conventional dietary studies of the target or closely related species (Gerwing et al., 2016), as well as ancillary information on morphology, behaviour and ecology, which may provide a basis to assess the plausibility of direct ingestion of unexpected taxa detected in samples. Another potential way to identify secondary consumption could be to look at the proportion of reads of each taxon and try to understand if it always occurs at a low proportion or not (Deagle et al., 2019). By filtering all taxa with <1% of the total number of reads of the corresponding PCR, one could expect that secondary consumption would disappear. However, in our study we observed that this is not always the case, with high number of reads obtained for some taxa that probably resulted from secondary ingestion. Nevertheless, detection resulting from secondary ingestion may not always be a problem, e.g., if the study aim is to know the entire intake of a given species, irrespective of whether it was ingested directly or indirectly (Pompanon et al., 2012).

Second, our study confirmed the value of using multiple markers, but suggests that previous studies based on a mix of nonoverlapping specific markers each targeting a particular dietary component (e.g., Coghlan et al., 2013; Groom et al., 2017; Robeson et al., 2018; Sullins et al., 2018) may not be sufficient to overcome marker biases and thus provide a reliable diet composition. This is because markers considered universal for a given taxonomic clade may still have considerable variations in affinity across taxa within that clade, and thus may not amplify some important items in the diet (Aizpurua et al., 2018; Alberdi et al., 2019, 2017; Bowser et al., 2013; Clarke et al., 2014; Kaunisto et al., 2017; Piñol et al., 2015). The problem was clearly illustrated by the high level of bias detected for ZBJ, which is sometimes regarded as universal for arthropods and is still the only marker used in many studies (Gordon et al., 2019; McClenaghan et al., 2019; Moran et al., 2019). In our study ZBJ completely missed Formicidae and other Hymenoptera, which was a key component of the diet identified through other methods, and probably overestimated the dietary importance of Lepidoptera and Diptera. The 16S marker used appeared less biased and thus may provide an alternative to ZBJ (Clarke et al., 2014; Deagle, Jarman, Coissac, Pompanon, & Taberlet, 2014), but it still underestimated some important dietary components, which may be partly due to the less comprehensive reference databases available when compared to COI (Elbrecht et al., 2016).

To overcome the problems of marker bias and taxonomic resolution, a mix of taxonomic data from different markers needs to be integrated, by eliminating the duplicates resulting from the same individual prey being detected at different taxonomic resolutions. This should be a relatively easy task using the criteria and the python script provided in our study. Notwithstanding, newer and better molecular markers have been developed and are now available, and these may reduce the need for a multi‐marker approach, e.g., UniPlant for plants (Moorhouse‐Gann et al., 2018) and fwh for insects (Vamos, Elbrecht, & Leese, 2017). Unfortunately, there are no perfect markers, thus multiple primer sets should most of the time detect more taxa, mainly in highly diverse groups such as invertebrates (Corse et al., 2019). Even though untested in this study, our script should also prove useful in any broadscale biodiversity assessment, using either eDNA or bulk samples, allowing the integration of taxa detected using any combination of molecular markers, as well as of taxa detected through other methods like morphological identification.

## AUTHOR CONTRIBUTIONS

The first and second author contributed equally to this paper. L.P.S., and V.A.M. designed the study with improvements from all authors. L.P.S., V.A.M., P.B.L., and R.J.L. collected the samples. V.A.M. performed the molecular analysis. L.P.S., and P.B.L. did the morphological analysis. P.P. developed the python script. L.P.S., V.A.M. and P.P. did the bioinformatic and statistical analysis. L.P.S., V.A.M., and P.B. led the writing with substantial contributions from all authors.

## Data Availability

Sequencing data, morphological and ESVs occurrence and identification are available on Dryad: https://doi.org/10.5061/dryad.26vr077 (da Silva et al., 2019). Merge_markers, the python script to combine the dietary data of different markers, is deposited on GitHub: https://github.com/PJADPereira/merge_markers
