# Supplementary material for: Advancing the integration of multi‐marker metabarcoding data in dietary analysis of trophic generalists
Source: Mol Ecol Resour. 2019 Aug 26;19(6):1420–32. doi: 10.1111/1755-0998.13060 (PMC6899665; doi:10.1111/1755-0998.13060)
Supplement: Supplementary file 1 [file MEN-19-1420-s001.pdf]

MOLECULAR ECOLOGY RESOURCES

Supplemental Information for:

Advancing the integration of multi-marker metabarcoding data in dietary analysis of trophic generalists

Luis P. da Silva, Vanessa A. Mata, Pedro B. Lopes, Paulo Pereira, Simon N. Jarman, Ricardo J. Lopes, Pedro Beja

Table of Contents:

**Figure S1**	Page 2
**Table S1**	Page 3
**Table S2**	Page 4
**Table S3**	Page 5

MOLECULAR ECOLOGY
RESOURCES

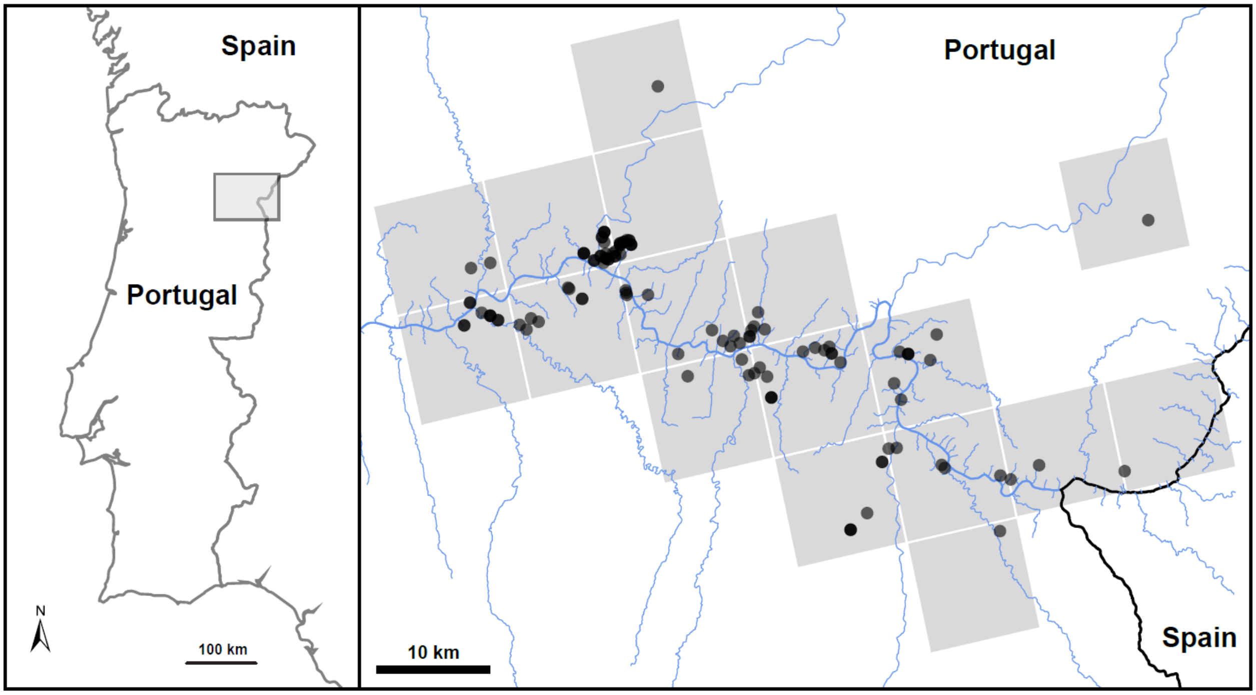

Figure S1 – Black Wheatear distribution along the Douro valley in Portugal (10 km^2 grey squares in ETRS89 projection) and the sample collection points.

MOLECULAR ECOLOGY
RESOURCES

Table S2 – Multiple comparison of taxa and order diversity among different diet assessment methods and molecular markers. Significant values are highlighted in bold.

	Group	Comparison	Estimate	Std. error	z-value	p-value
No. Taxa	Plant	morph – 18S	-1.0986	0.1308	-8.397	**<0.001**
		morph – *trn*L	1.8867	0.1216	15.517	**<0.001**
		18S – *trn*L	0.7881	0.0789	9.988	**<0.001**
		multi – 18S	0.8979	0.0772	11.631	**<0.001**
		multi – *trn*L	-0.1099	0.0604	-1.818	0.207
	Animal	morph – 18S	0.44944	0.08692	5.171	**<0.001**
		morph – IN16STK	-0.48801	0.06891	-7.082	**<0.001**
		morph – ZBJ	0.15373	0.07393	2.079	0.225
		18S – IN16STK	0.93745	0.08011	11.701	**<0.001**
		18S – ZBJ	0.60317	0.08447	7.141	**<0.001**
		IN16STK – ZBJ	-0.33429	0.0658	-5.081	**<0.001**
		multi – 18S	1.4240	0.0755	18.867	**<0.001**
		multi – IN16STK	0.4865	0.0539	9.029	**<0.001**
		multi – ZBJ	-0.8208	0.0601	-13.647	**<0.001**
No. Orders	Plant	morph – 18S	-1.02217	0.13287	-7.693	**<0.001**
		morph – *trn*L	1.66501	0.12425	13.4	**<0.001**
		18S – *trn*L	0.64284	0.08442	7.614	**<0.001**
		multi – 18S	0.7521	0.0823	9.137	**<0.001**
		multi – *trn*L	-0.1093	0.0678	-1.613	0.320
	Animal	morph – 18S	0.08791	0.10176	0.864	1.000
		morph – IN16STK	-0.49385	0.08928	-5.531	**<0.001**
		morph – ZBJ	0.32294	0.09238	3.496	**0.003**
		18S – IN16STK	0.58176	0.0918	6.338	**<0.001**
		18S – ZBJ	0.41086	0.09481	4.333	**<0.001**
		IN16STK – ZBJ	-0.17091	0.08127	-2.103	0.212
		multi – 18S	1.0411	0.0853	12.210	**<0.001**
		multi – IN16STK	0.4594	0.0700	6.562	**<0.001**
		multi – ZBJ	-0.6303	0.0739	-8.530	**<0.001**

MOLECULAR ECOLOGY RESOURCES

Table S3 - Multivariate Generalized Linear Models univariate comparisons among plant orders. Significant values are highlighted in bold.

	morphological vs markers		markers vs multi-marker	
Order	Deviance	p-value	Deviance	p-value
Asparagales	2.206	0.603	1.631	0.985
Poales	19.934	**0.001**	12.749	**0.027**
Apiales	9.731	**0.040**	0.388	0.985
Asterales	101.287	**0.001**	18.261	**0.003**
Boraginales	2.449	0.603	0.553	0.985
Brassicales	14.518	**0.004**	4.999	0.740
Caryophyllales	34.946	**0.001**	34.999	**0.001**
Dipsacales	4.394	0.418	3.243	0.940
Ericales	4.394	0.418	3.243	0.940
Fabales	20.673	**0.001**	4.637	0.740
Fagales	20.334	**0.001**	16.171	**0.006**
Gentianales	6.655	0.149	4.93	0.740
Geraniales	6.591	0.159	4.865	0.740
Lamiales	57.754	**0.001**	31.347	**0.001**
Malpighiales	14.461	**0.004**	7.305	0.324
Malvales	9.749	**0.040**	2.657	0.940
Myrtales	2.197	0.603	1.622	0.985
Ranunculales	4.394	0.418	3.243	0.940
Rosales	23.359	**0.001**	2.894	0.940
Santalales	5.982	0.192	2.717	0.940
Sapindales	10.996	**0.024**	1.725	0.985
Saxifragales	27.293	**0.001**	20.401	**0.001**
Solanales	6.239	0.192	7.089	0.327
Vitales	108.044	**0.001**	4.702	0.740
Zygophyllales	2.197	0.603	1.622	0.985
Pinales	17.401	**0.002**	9.151	0.137
Polypodiales	6.591	0.149	4.865	0.740

MOLECULAR ECOLOGY RESOURCES

Table S4 – Multivariate Generalized Linear Models univariate comparisons among animal orders. Significant values are highlighted in bold.

	morphological vs markers		markers vs multi-marker	
Order	Deviance	p-value	Deviance	p-value
Acariformes	2.772	0.91	2.772	0.979
Araneae	23.783	**0.002**	41.289	**0.001**
Sarcoptiformes	11.09	0.062	11.09	0.143
Trombidiformes	13.862	**0.024**	13.862	**0.030**
Lithobiomorpha	8.38	0.239	8.401	0.413
Scolopendromorpha	2.772	0.896	1.53	0.979
Haplotaxida	2.772	0.910	2.772	0.979
Julida	11.228	0.054	9.933	0.229
Pulmonata	2.772	0.910	2.772	0.979
Archaeognatha	8.858	0.228	7.584	0.519
Blattodea	0.541	0.950	0.68	0.979
Coleoptera	12.934	**0.040**	37.884	**0.001**
Dermaptera	8.317	0.239	7.022	0.592
Diptera	97.8	**0.001**	130.311	**0.001**
Embioptera	22.18	**0.002**	22.18	**0.001**
Hemiptera	18.272	**0.003**	34.36	**0.001**
Hymenoptera	190.467	**0.001**	180.358	**0.001**
Isoptera	5.545	0.557	5.623	0.787
Lepidoptera	160.152	**0.001**	138.303	**0.001**
Mantodea	8.72	0.239	5.094	0.864
Neuroptera	8.538	0.239	4.232	0.977
Odonata	8.317	0.239	8.317	0.413
Orthoptera	78.267	**0.001**	52.062	**0.001**
Phasmatodea	2.772	0.910	1.726	0.979
Psocoptera	2.772	0.910	2.772	0.979
Zygentoma	4.498	0.851	3.314	0.979
Isopoda	21.577	**0.003**	19.208	**0.001**
Squamata	9.445	0.151	11.255	0.125
